# Incidence and cost estimate of treating pediatric adverse drug reactions in Lagos, Nigeria

**DOI:** 10.1590/S1516-31802011000300006

**Published:** 2011-05-05

**Authors:** Kazeem Adeola Oshikoya, Henry Chukwura, Olisamedua Fidelis Njokanma, Idowu Odunayo Senbanjo, Iyabo Ojo

**Affiliations:** I MD, MSc. Lecturer, Pharmacology Department, Lagos State University College of Medicine, and Resident, Paediatrics Department, Lagos State University Teaching Hospital, Ikeja, Lagos, Nigeria.; II BPharm, MPH. Assistant Director of Pharmacy, Pharmacy Department, Lagos State University Teaching Hospital College of Medicine, Ikeja, Lagos, Nigeria.; III MD, FWACP, FMCPaed. Professor and Head, Paediatrics Department, Lagos State University Teaching Hospital, Ikeja, Lagos, Nigeria.; IV MD, FWACP. Consultant Pediatrician, Paediatrics Department, Lagos State University Teaching Hospital, Ikeja, Lagos, Nigeria.; V BPharm. Senior Pharmacy Superintendent, Pharmacy Department, Lagos State University Teaching Hospital College of Medicine, Ikeja, Lagos, Nigeria.

**Keywords:** Drug toxicity, Child, Hospitalization, Body burden, Nigeria, Toxicidade de drogas, Criança, Hospitalização, Carga corporal (Radioterapia), Nigéria

## Abstract

**CONTEXT AND OBJECTIVES:**

Adverse drug reactions (ADRs) may cause prolonged hospital admissions with high treatment costs. The burden of ADRs in children has never been evaluated in Nigeria. The incidence of pediatric ADRs and the estimated cost of treatment over an 18-month period were determined in this study.

**DESIGN AND SETTING:**

Prospective observational study on children admitted to the pediatric wards of the Lagos State University Teaching Hospital (LASUTH) in Nigeria, between July 2006 and December 2007.

**METHODS:**

Each patient was assessed for ADRs throughout admission. Medical and non-medical costs to the hospital and patient were estimated for each ADR by reviewing the medical and pharmacy bills, medical charts and diagnostic request forms and by interviewing the parents. Cost estimates were performed in 2007 naira (Nigeria currency) from the perspectives of the hospital (government), service users (patients) and society (bearers of the total costs attributable to treating ADRs). The total estimated cost was expressed in 2007 United States dollars (USD).

**RESULTS:**

Two thousand and four children were admitted during the study; 12 (0.6%) were admitted because of ADRs and 23 (1.2%) developed ADR(s) during admission. Forty ADRs were suspected in these 35 patients and involved 53 medicines. Antibiotics (50%) were the most suspected medicines. Approximately 1.83 million naira (USD 15,466.60) was expended to manage all the patients admitted due to ADRs.

**CONCLUSIONS:**

Treating pediatric ADRs was very expensive. Pediatric drug use policies in Nigeria need to be reviewed so as to discourage self-medication, polypharmacy prescription and sales of prescription medicines without prescription.

## RESUMO

**CONTEXTO E OBJETIVOS:**

Reações adversas a drogas (RAD) podem causar hospitalização prolongada com alto custo de tratamento. Este efeito das RAD em crianças nunca foi avaliado na Nigéria. A incidência de RAD em pediatria e o custo estimado de tratamento em um período de 18 meses foram determinadas neste estudo.

**DESENHO E LOCAL:**

Estudo prospectivo observacional de crianças admitidas nas unidades pediátricas do Hospital Universitário do Estado de Lagos (LASUTH) na Nigéria, entre julho de 2006 e dezembro de 2007.

**MÉTODOS:**

Cada paciente foi avaliado para RAD durante a admissão. Os custos médicos e não médicos para o hospital e para o paciente foram estimados para cada RAD pela revisão das contas médicas e da farmácia, pelos registros médicos, pelos exames necessários para diagnóstico e pelas entrevistas com os pais. Os custos foram estimados em nairas (moeda nigeriana) de 2007 a partir das perspectivas do hospital (governo), dos usuários de serviços (pacientes) e da sociedade (portadores dos custos totais atribuídos ao tratamento de RAD). A estimativa de custo total foi apresentada em dólares americanos (US) de 2007.

**RESULTADOS:**

Duas mil e quatro crianças foram admitidas durante este estudo; 12 (0,6%) foram admitidas devido a RAD e 23 (1.2%) desenvolveram RAD durante admissão. Quarenta RAD foram suspeitadas nesses 35 pacientes e envolveram 53 medicamentos. Antibióticos (50%) foram as drogas mais suspeitas. Aproximadamente 1.83 milhões de nairas (US 15,466.60) foram gastos para cuidar de todos os pacientes admitidos por RAD.

**CONCLUSÕES:**

O tratamento das RAD pediátricas foi bastante caro. As políticas de uso de drogas em pediatria na Nigéria devem ser revistas para desencorajar a auto-medicação, a prescrição de polifarmácia e a venda de medicamentos de prescrição sem prescrição.

## INTRODUCTION

Adverse drug reactions (ADRs) occur frequently and globally, accounting for a significant number of fatalities each year.^1,2^ It has been estimated that fatalities directly attributable to ADRs are the fourth to sixth leading cause of death in hospitals in the United States, exceeding deaths caused by pneumonia and diabetes.^3^ Among a population of adults in Cameroon, the rate of ADRs was 3.5%^4^ and among adult medical inpatients in South Africa, the rate was 12.6%.^5^ There is a relative dearth of epidemiological studies of ADRs in pediatric patients in Nigeria and other African countries. Previous studies have reported ADR rates of 2.1% to 9.5% in pediatric populations.^6,7^ However, these studies were conducted in developed countries where disease patterns, access to medicines, medicine use patterns and patient management differ significantly from those of developing countries.^8^ We have reported an ADR rate of 1.2% among pediatric patients in Nigeria.^2^

The financial burden resulting from medicine-related morbidity and mortality is equally significant and has been conservatively estimated as United States dollars (USD) 30 billion annually in the United States.^9^ Deaths resulting from ADRs have been reported in Nigeria.^2^ In addition to the human costs, ADRs have a major impact on public health by imposing a considerable financial burden on society and the already-stretched healthcare systems. In Spain, the minimum direct cost to the public health system of diagnosing and treating adult patients with suspected ADRs in a single month was estimated as 42,732 Ecus (USD 54299.57).^10^ Wu and Pantaleo^11^ estimated the mean cost of treating an ADR per patient as USD 9,491 with 50% of this cost being the hospitalization or room charges alone. In India, Patel et al.^12^ also estimated the total cost to a hospital due to hospitalization of patients with ADRs over a six-week period in an emergency room as USD 27,358.

Although nearly all medicines are capable of causing injuries, certain medicines such as antibiotics,^7,13^ immunosuppressive agents^14^ and anticonvulsants^13,14^ are more likely to be associated with ADRs in children. Antibiotics and sulfadoxine/pyrimethamine antimalarials are the leading cause of ADRs in children in Nigeria,^2^ since they constitute the medicines most frequently self-medicated to children,^15^ most regularly prescribed by doctors^15,16^ and most frequently stocked at home by parents.^15^

Prevention of medicine-related morbidity and mortality has become an increasingly important requirement for reducing the morbidity, mortality and healthcare expenditure relating to ADRs. Estimates of treatment costs in hospital cost-effectiveness studies need to take the cost of medicine into consideration, but this should not be limited to its purchase price.^17^ The real cost of a medicine must also take into account the impact of ADRs potentially induced by this medicine.^18^ Despite the widespread impression that ADRs in hospitalized patients are costly because they require additional treatments and prolong the length of stay,^19^ the exact cost attributed to ADRs has not been studied in children.

The various risk factors associated with ADRs in children and the likely medicines involved have previously been reported in Nigeria.^2^ However, lack of national pediatric pharmacovigilance schemes makes information collation about ADRs and its reporting nationally poor, such that ADRs remain a clinical problem. A few studies have examined the clinical and epidemiological trends of ADRs in Nigerian children,^2^ but none have specifically evaluated the cost of care associated with them.

## OBJECTIVE

The aims of this study were to determine the incidence of ADRs as a cause of pediatric admissions at the Lagos State University Teaching Hospital (LASUTH) over an 18-month period and to estimate the cost of treating these patients. The incidence of ADRs in pediatric inpatients, after hospital admission, was also determined over the study period.

## PATIENTS AND METHODS

### Patients and settings

This prospective observational study was conducted at LASUTH in Nigeria and involved children admitted because of suspected ADRs, or who developed ADRs while on admission, in a 98-bed children's ward between July 2006 and December 2007. Patients admitted for less than 24 hours or with repeated admissions, and those whose medical records were unavailable for review, either during the admission or following discharge, were excluded from the study.

Lagos is the smallest but most populous state in Nigeria, with an estimated population of 15 million according to the 1991 national census. Both public and private healthcare systems are practiced in Nigeria. Primary health centers, general hospitals, federal medical centers and teaching hospitals constitute the public healthcare system; while individually owned hospitals and clinics constitute the private healthcare system. Lagos is served by two teaching hospitals: the Lagos University Teaching Hospital (LUTH) and Lagos State University Teaching Hospital (LASUTH). While LUTH is funded by the Federal Government, LASUTH is funded by the Lagos State Government. The facilities available and standard of care obtainable from the two hospitals are comparable. Of the two hospitals, pediatric health care is partly free at LASUTH; the cost of treating pediatric inpatients is borne partly by the Lagos State Government and partly by parents. Two pharmacies are in operation at LASUTH; the non-fee paying pharmacy (where antimalarials for all, medicines and other health products for children and the elderly are available free of charge) and the fee-paying pharmacy (a private venture that sells other medicines and health products for children and adults that are unavailable at the non-fee-paying pharmacy). Hospitalization, provider services and diagnostic investigations are free for pediatric and elderly patients at LASUTH. Parents are allowed to have investigations done that were unavailable in the hospital elsewhere without reimbursement. Also, medicines unavailable in the non-fee-paying pharmacy were either procured from the hospital fee-paying pharmacy or from community pharmacies. Since children neither pay for hospitalization nor pay for provider services at LASUTH, we decided to use the pediatric cost of hospitalization and provider services from LUTH. The cost of specific investigations done outside the hospital and the costs of medications obtained from both the fee-paying pharmacy and community pharmacies were also used to calculate the medical expenditures. The study therefore estimated the financial burden of ADRs from the perspectives of the service provider (government), service users (patients) and society (bearers of the total costs attributable to treating ADRs).

### Patient evaluation and data collection on ADRs

During the study period, a research team comprising a pediatric clinical pharmacologist, pediatricians and two hospital pharmacists, who were members of the ADR monitoring committee of LASUTH, prospectively assessed all admissions to the pediatric wards to determine whether patients had been admitted as a result of a suspected ADR or whether an ADR had occurred after admission. All the members of the research team had been trained many times on how to recognize, assess and report suspected ADRs. The team members had also acquired additional experience from participating in previous studies on pediatric ADRs.^2^

On each day of the study period, a specific questionnaire was completed for all children newly admitted. All children were evaluated daily for the presence of ADRs, by the research team, and were observed until discharge to ascertain the final diagnosis. Patient evaluation was usually carried out about two hours before the normal ward rounds. The evaluation consisted of examining medical and nursing records, reviewing prescription charts and attending clinical rounds. All the pediatricians, junior doctors and nurses were asked to participate in the study and to report or record any suspected ADR.

ADRs were defined in accordance with Edwards and Aronson.^20^ This definition excludes ADRs that require no intervention and has been applied in a previous ADR study on adults.^21^ If a suspected ADR was reported, the research team usually evaluated all the suspected cases. The pediatric clinical pharmacologist and the more senior of the two pharmacists assisted in assessing the suspected ADRs for causality, severity and avoidability. ADRs were identified on the basis that they were well recognized as shown by their inclusion in the summary of product characteristics,^22^ the Nigerian National Drug Formulary^23^ and the pediatric British National Formulary,^24^ or in previously published case reports. ADRs were also classified as type A (dose dependent and predictable from known pharmacology) or type B (idiosyncratic, no clear dose response and not predictable from the known pharmacology), in accordance with the classification of Rawlins and Thompson.^25^

Data on the particular suspected medicines and reactions were collected in a suitably designed ADR documentation form. All relevant data, including all medicines the patient had received before the onset of the reactions, their respective doses, the routes of administration with their frequency, laboratory test results present in the medical records, clinical details (system-organ class involvement) and the treatment (pharmacological and non-pharmacological), were noted. In addition, where the documented medicine history was unclear, the patient's medication history was taken from the patients or parents/guardians or the attending physicians, and comorbidity was identified to assess the causal relationship between the suspected medicine and the reaction. Information regarding previous medicine use was obtained by interviewing parents, relatives, nurses, or others, as necessary. A modified list of trigger events requiring further assessment for medicine-related causes (Table 1) was adapted from the work of Rozich et al.,^26^ to increase the sensitivity to possible ADRs on admission. All the members of the research team familiarized themselves with the list, and the document was used as a pocket reference. In situations in which an identical reaction occurred more than once in the same patient during the same hospital stay, the patient was documented as having experienced a single reaction.

**Table 1.** List of triggers and process identified in children with adverse drug reactions (ADR)

Trigger	Process identified
Diphenhydramine, promethazine, or/and any other antihistamine	Hypersensitivity reaction or drug effect
Systemic or localized topical corticosteroid*	Hypersensitivity reaction
Vitamin K or fresh frozen plasma*	Over-anticoagulation with warfarin or drug-induced DIC
Metoclopramide or other antiemetic*	Nausea or emesis related to drug use
Antidiarrheals	Drug-induced diarrhea
Sodium polystyrene	Hyperkalemia from renal impairment/drug effect
Insulin with glucose*	Hyperkalemia from renal impairment/drug effect
50% dextrose	Hypoglycemia (possibly with insulin)
Flumazenil	Oversedation with benzodiazepines
Naloxone	Oversedation or respiratory depression with narcotics
Phenobarbitone statim or ≤ 24 hours*	Drug-induced seizure
Diazepam stat or ≤ 24 hours*	Drug-induced seizure
Adrenalin	Anaphylaxis /bronchospasm
Biperiden*	Extrapyramidal effect to phenothiazine
Atropine*	Extrapyramidal effect to phenothiazine
Dopamine*	Drug-induced hypotension
Cimetidine or other anti-ulcer agents*	Drug-induced gastritis
Laboratory triggers	
Potassium < 3.5 mmol/l	Hypokalemia due to loop diuretics
PTT > 100 seconds	Over-anticoagulation with heparin
WBC < 3000 × 10^6^/µl	Neutropenia due to drug or disease
ALT > thrice the normal*	Hepatotoxicity (possibly due to drug)
Bilirubin > twice the normal*	Hepatotoxicity (possibly due to drug)
Serum glucose ≤ 2.5 mmol/l	Hypoglycemia due to insulin
Rising level of serial serum creatinine	Drug-induced renal insufficiency
Event triggers	
Oversedation, lethargy or fall	Related to overuse of medication
Skin rash or ulceration	Drug-related adverse event
Lip swelling and/or angioedema*	Drug-related adverse event
Seizures and/or dizziness*	CNS adverse drug event, drug toxicity
Dystonia, ataxia, torticollis and/or dyskinesia*	CNS adverse drug event, drug toxicity
Altered level of consciousness (after excluding cerebral malaria)*	CNS adverse drug event, drug toxicity
New bradycardia or tachycardia*	Drug-related cardiac event
New onset of jaundice*	Drug-related hepatotoxicity
New hypotension (defined by BP values relative to age)*	Drug-related vascular event
New cardiac failure*	Drug-related cardiotoxicity
Wheezing*	Allergic reaction
Abrupt medication stop	Adverse event
Transfer to PICU in another hospital*	Adverse event
Event suspected to be drug-related* by doctor, nurse or parents	Suspected ADR

* Event added or modified from trigger event published by Rozich et al.^26^. Modification was based on pattern of drug therapy used at the Lagos State University Teaching Hospital and the available essential medicines in Nigeria. Some modifications were made to suit pediatric age group. DIC = disseminated intravascular coagulopathy; PTT = partial thromboplastin time; WBC = white blood cell count; ALT = alanine transaminase; PICU = pediatric intensive care unit; CNS = central nervous system. Serum toxic levels of drugs were excluded.

The suspected ADRs and the suspected medicines were classified in accordance with the World Health Organization (WHO) classification.^27^ The causality relationship between the suspected ADR and the suspected medicine therapy was assessed case by case using the Jones method.^28^ Severity was classified according to the Schirm et al.^29^ classification for pediatric age group: fatal (resulting in near death or eventual death for the patient); severe (directly life-threatening and/or prolonged hospitalization, associated with organ or system dysfunction and permanent harm); moderate (requiring treatment intervention, and patient temporarily harmed); and mild (uncomplicated primary disease, increased patient monitoring, no treatment required and drug discontinuation not necessary). Avoidability was assessed within the local context of medicine management for the patient. A reaction was considered to be "avoidable" if it met the criteria of Schumock and Thornton,^30^ i.e. the medicine involved in the ADR was inappropriate for the patient's age, weight and disease state; the dose, route or frequency of administration were inappropriate for the patient's age, weight and disease state; required therapeutic drug monitoring or other necessary laboratory tests were not performed; past history of allergy or previous reactions to the medicine; documented toxic serum drug level; and deviation from the recommended dose.

Analysis for causality, severity and avoidability was done independently by two of the investigators (KAO and HAC). Overall, there was approximately 70% agreement in the causality and avoidability assessments, using the kappa statistics. Any discrepancies were then discussed with other investigators to achieve a consensus.

The length of stay was calculated separately for each admission and was used to determine the total number of bed days and the mean length of stay. The ADR admission rate was determined based on the number of patients admitted at least once with an ADR during the study.

### Health cost of ADRs

Only the 12 patients admitted because of ADRs were evaluated for the health cost of ADRs. We excluded the patients who developed ADRs while on admission, because they had not fully recovered from the primary disease for which they were admitted. It was also difficult to ascertain whether prolonged hospitalization was due to the primary disease or to the ADRs. Only the cost of treating moderate and severe ADRs (including the two fatal cases) was estimated, since most cases of mild ADRs were treated at the outpatient clinic.

Admissions due to an ADR were evaluated for the total length of hospitalization as well as the direct and indirect costs of treatment. Any case that did not involve stopping the offending medicine or continuation of treatment without any change was considered as nil. The costs of managing an ADR were determined as highlighted below. The total estimated cost of treating ADRs was expressed in United States dollars (USD) of 2007, at a rate of 1 USD equals 118 naira (₦) (i.e. 2007 naira values).

#### Direct medical costs to the hospital

The medical and pharmacy bills, as well as medical charts, were reviewed for each patient admitted due to ADRs, in order to document all medications received by the patient and the diagnostic tests associated with ADRs. Medicines bought at the hospital pharmacy or from community pharmacies, as well as investigations done outside the hospital, were excluded at this stage.

ADR management was symptomatic and varied according to severities and the organ or system involved. Thus, a wide variety of medications and diagnostic investigations were involved in the management. Unit costs were therefore calculated for medications and diagnostics associated with each case of ADRs, as well as the costs for hospital bed-days for each patient. Medications were assigned wholesale medicine costs per unit, based on the LASUTH fee-paying pharmacy prices, while the laboratory manager provided pre-calculated costs for each diagnostic test. The total quantity of each medication used per patient multiplied by its unit cost, and the total number of specific diagnostic investigations performed on each patient multiplied by the unit cost were summed to yield the direct medical cost to the hospital incurred per patient. The total direct medical cost to the hospital incurred by all the patients is presented in Table 2. Ideally, patients with severe ADRs were supposed to be nursed in a pediatric intensive care unit (PICU) but such a facility was not available in the hospital; therefore, we did not include the cost of PICU. The total medication and diagnostic costs to the healthcare system equaled the sum of the individual unit costs multiplied by the quantity of medications and diagnostic tests ordered, respectively.

**Table 2.** Mean cost to the hospital per single adverse drug reaction (ADR) admission in 2007 naira (₦)

Direct medical costs to hospital(government)	Moderate (n = 5)	Severe (n = 7)	P-value
Days of hospitalization	10.4 ± 2.1 days	15.7 ± 7.4 days	< 0.001
Hospitalization costs*	₦ 2,080.00 ± ₦ 420.00	₦ 3,140.00 ± ₦ 680.00	0.043
Healthcare professional fees†	₦ 4,308.72 ± ₦ 870.03	₦ 13,233.53 ± ₦ 3,625.00	< 0.001
Medication costs‡	₦ 3,400.00 ± ₦ 240.00	₦ 36,800.00 ± ₦ 7,200.00	< 0.001
Diagnostic costs§	₦ 3,300.00 ± ₦ 232.10	₦ 10,750.00 ± ₦ 2,788.00	< 0.001
Others||	₦ 600.00 ± ₦ 59.20	₦ 1,800.00 ± ₦ 498.00	< 0.001
	₦ 13,688.72 ± ₦ 1,821.33	₦ 65,723.53 ± ₦ 14,791.00	< 0.001
Follow up visit costs¶	₦ 1,628.60 ± ₦ 61.28	₦ 6,170.00 ± ₦ 1,729.10	< 0.001
Total cost per ADR case:			
Naira	15,317.32 ± 1,882.61	71,893.53 ± 16,520.10	< 0.001
USD (1 USD = ₦ 118)	129.81 ± 15.95	609.27 ± 140.00	

n = number of patients with ADRs;

* the bed costs during admission;

† the doctors’ and paramedics’ fees for managing the patient during admission;

‡ the costs of medicines obtained free of charge from the non-fee-paying pharmacy;

§ the costs of investigation done free of charge during hospital admission;

|| Others refers to the mean cost of electricity, linen, water supplies, building and equipment depreciation, maintenance and medical record expenses incurred by the healthcare facility with inpatient expenses calculated at an 80% occupancy rate;

¶ medication, diagnostic and procedure costs rendered free of charge to the patients during follow up visit; ₦: 2007 naira (Nigerian currency).

The hospital care and bed-day costing included staff salaries per day, electricity, linen, water supplies, building and equipment depreciation, maintenance and medical record expenses incurred by the healthcare facility, with inpatient expenses calculated at 80% occupancy rate. Follow-up visits, after discharge, were arranged so that the patients’ relative stability could be monitored. The actual charge for any prior visit before the appointment dates or subsequent consultation with any general or specialist doctors and other paramedics within the hospital were included in the total visit cost.

The direct medical costs to the hospital equaled the sum of all medication, diagnostic and visit costs. The direct medical cost incurred in managing patients admitted because of ADRs was calculated as the total amount spent by the hospital on the patients divided by the total number of patients admitted due to ADRs.

#### Direct medical costs to the patient

This refers to any bill incurred by the patient on medications, diagnostic investigations or medical procedures while on admission or during follow-up visits. This was recorded for all medications procured at the fee-paying pharmacy and community pharmacies, and for all diagnostic tests associated with ADRs that were done outside the hospital. This was achieved by reviewing all the prescriptions, medical bills and charts in the case file, and by interviewing the parents. Patient-reported costs of outside private practitioner visits at the onset of ADRs and for follow-up were included. The direct medical cost to the patient therefore equaled the sum of previous and present admissions due to ADRs, medication and diagnostic costs.

#### Direct non-medical costs

The direct non-medical costs equaled the sum of all transportation, food and hotel expenses incurred during the hospital admission for ADRs since some of the patients were referred from distant health centers.

#### Indirect costs

The indirect costs of the ADRs per household refer to the wages lost by each parent caused by nursing a child with an ADR. These were calculated by using the human capital method, which measures the parents’ average monthly wages. The indirect cost equaled the sum of each parent's estimated daily wage based on the minimum monthly wage (7,500 naira of 2007) for workers in Lagos multiplied by lost workdays. An individual employee in Lagos would work for an average of 9 hours per day or 45 hours per week. Each parent who spent up to 9 hours or more per day with an ADR patient was considered to have lost a day of work.

### Ethical issues and statistical analyses

The study protocol was approved by the ethics committee of LASUTH. All data from the questionnaires and the medical records were coded and statistical analysis on the results was performed using the Statistical Package for the Social Sciences (SPSS), version 13. Comparisons between ADR and non-ADR patients with regard to continuous data were made using the t-test or the Mann-Whitney U-test, depending on skewness, and the chi-square test was used for comparisons of continuous data between ADR patients at a significance level of P < 0.05.

## RESULTS

### Demographic data on the patients and their parents

Two thousand and four children were admitted during the 18-month prospective study, of whom 1222 (61%) were males. The admission rate was 111 per month. Twelve patients (0.6%) were admitted due to ADRs, and 23 inpatients (1.2%) developed ADRs, thus giving an overall incidence of 1.8%. Male patients experienced significantly more ADRs (23; 65.7%) than female patients (12; 34.3%; P = 0.02) but there was no significant gender difference between the ADR and non-ADR patients (male-to-female ratios of 1.92 and 1.50, respectively) (P = 0.08). The mean age of the patients admitted due to ADRs was 8.9 ± 3.4 years (age range, 3-12 years) and was not significantly different from the mean age of the inpatients who developed ADRs (5.1 ± 3.8 years; range, 2 days-12 years) (P = 0.81). The overall mean age of the ADR patients (5.0 ± 3.2 years) was not significantly different from the mean age of the non-ADR patients (6.4 ± 4.1 years) (P = 0.72).

The mothers’ education levels, their occupations, annual household income (in naira) and costing data for the hospital are presented in Table 3.

**Table 3.** Baseline demographics of the mothers and costing data for the hospital (Lagos State University Teaching Hospital, LASUTH)

Parameters	Moderate (n = 21)	Severe (n = 14)	P-value
Mothers’ demographics			
Mean age (years)	36.5 ± 6.8 years	37.2 ± 5.5 years	0.873
Levels of education			
None	3	2	0.527
Primary	7	4	0.865
Secondary	8	6	0.804
University/polytechnic/college	3	2	0.527
Mean annual household income	₦ 210,825.00 ± ₦ 15,225.70	₦ 186,608.00 ± ₦ 32,318.44	0.693
Hospital costs			
Inpatient cost per bed day	₦ 200.00	₦ 200.00	-
Pediatrician consultation fee	₦ 214.30	₦ 214.30	-
Specialist consultation fee	₦ 214.30	₦ 214.30	-
Nursing care fee	-	-	-
Social worker fee	₦ 100.00	₦ 100.00	-
Psychologists fee	₦ 100.00	₦ 100.00	-
*Other cost per day	-	-	-

* Other cost refers to the mean cost of electricity, linen, water supplies, building and equipment depreciation, maintenance and medical record expenses incurred by the healthcare facility with inpatient expenses calculated at an 80% occupancy rate.

n = number of patients with adverse drug reactions (ADRs); ₦ = naira.

The patients were admitted because of a variety of diseases, but mainly malaria (842; 42%) and meningitis (365; 18.2%). A total of 8,417 medicines were received by the 2,004 patients (4.2 medicines per patient; range, 2-10). The most frequently used medicines were cefuroxime and gentamicin. The inpatients who developed ADRs were prescribed a total of 67 medicines, including multivitamins, hematinics and antioxidants (mean of 4.5 medicines per patient; range 4-8). There was no significant difference in the mean number of medicines received between the inpatients who developed ADRs (4.5 medicines per patient) and the non-ADR inpatients (4.1 medicines per patient) (P = 0.08).

### ADRs detected, drugs incriminated and organs or systems affected

Table 4 summarizes the ADRs detected according to the organ or system affected. Cutaneous manifestation (17; 43%) was the commonest means. Tables 5 and 6 show the pattern of ADRs and the suspected medicines recorded during the study period. Erythema multiforme rash (7) and pustular rash (4) were the commonest clinical manifestations. In all, 53 medicines were suspected of causing 40 ADRs (Table 4). Half of the medicines were antibiotics.

**Table 4.** Systemic-organ classes of adverse drug reactions (ADRs) and the suspected medicines

Types of reaction	Suspected medicines*	Number of ADRs
Cutaneous manifestation		
Erythema multiforme	Co-trimoxazole (4), Ampicillin (2), Sulfadoxine/pyrimethamine (1), Phenobarbitone (2), Cefixime (1)	7
Pustular rash	Vancomycin (4)	4
Macular and morbiliform rash	Ampicillin (1), Albendazole (1), Cefotaxime (1)	3
Stevens-Johnson syndrome	Sulfadoxine/pyrimethamine (2), Co-trimoxazole (2), Ampicillin (2)	2
Angioneurotic edema	Amodiaquine (1)	1
Systemic manifestation		
Electrolyte disturbances	Prednisolone (2), Frusemide (2)	3
Red man syndrome	Vancomycin (2)	2
Anaphylactic shock	Ceftriaxone (1), Etoposide (1)	2
Hypothermia	Cefixime (1), Amodiaquine (1), Artesunate (1)	1
Tachycardia	Salbutamol (1)	1
CNS manifestation		
Dystonia	Amodiaquine (1), Artesunate (1), Promethazine (1), Cefixime (1)	2
Transient loss of vision	Quinine (1)	1
Seizure	Cefixime (1)	1
Sedation	Pentazocine	1
Endocrine manifestation		
Hyperglycemia	Prednisolone (1)	1
GIT manifestation		
Constipation	Metronidazole (1), Amoxicillin (1), Pentazocine (1), Tramadol (1)	3
Diarrhea	Amoxicillin/clavulanic acid (1)	1
Ileus	Loperamide (1), Frusemide (1)	2
Hematological manifestation		
Anemia	Carbamazepine (1)	1
Hemolysis	Co-trimoxazole (1)	1
Total	53	40

* more than one medicine was suspected for a single adverse reaction; CNS = central nervous system; GIT = gastrointestinal tract.

**Table 5.** Clinical details and characteristics of patients admitted due to adverse drug reactions and the suspected medicines (ADR)

Adverse drug reactions	Indication for the medicine	Medicine(s) used	Suspected medicine(s)	Age (years)	Sex	Severity	Duration of admission (days)
Dystonia	Malaria, enteric fever*	Cefixime, amodiaquine/artesunate, paracetamol	Amodiaquine, artesunate, cefixime	12	Male†	Moderate	4
Hypothermia	Malaria, enteric fever*	Cefixime, amodiaquine/artesunate, paracetamol	Amodiaquine, artesunate, cefixime	12	Male†	Moderate	4
Electrolyte disturbance	Status asthmaticus*	Prednisolone, salbutamol, erythromycin	Prednisolone	6	Female‡	Moderate	14
Hyperglycemia	Status asthmaticus*	Prednisolone, salbutamol, erythromycin	Prednisolone	6	Female‡	Moderate	14
Erythema multiforme	Generalized seizure*	Phenobarbitone	Phenobarbitone	6	Male	Severe	8
Erythema multiforme	URTI, malaria§	Sulfadoxine/pyrimethamine, co-trimoxazole, paracetamol	Sulfadoxine/pyrimethamine, co-trimoxazole	10	Male	Severe	21
Erythema multiforme	URTI||	Co-trimoxazole	Co-trimoxazole	12	Male	Severe	28
Erythema multiforme	URTI||	Co-trimoxazole, ampicillin	Co-trimoxazole, ampicillin	8	Female	Severe	18
Erythema multiforme	URTI, malaria§	Co-trimoxazole, chloroquine, paracetamol	Co-trimoxazole	10	Male	Severe	20
Macular and morbiliform rash	URTI¶	Ampicillin	Ampicillin	2	Female	Moderate	7
Macular and morbiliform rash	Abdominal pain§	Albendazole	Albendazole	10	Male	Moderate	10
Ileus	Diarrhea¶	Loperamide, co-trimoxazole, chloroquine	Loperamide	3	Male	Moderate	7
Stevens-Johnson syndrome	Malaria, enteric fever§	Ampicillin, co-trimoxazole, sulfadoxine/pyrimethamine, ibuprofen	Ampicillin, Co-trimoxazole, sulfadoxine/pyrimethamine	10	Male	Fatal	Died 10 days later
Stevens-Johnson syndrome	Malaria, URTI§	Ampicillin, co-trimoxazole, sulfadoxine/pyrimethamine	Ampicillin, Co-trimoxazole, sulfadoxine/pyrimethamine	12	Male	Fatal	Died 5 days later

* Medicine prescribed at the Lagos State University Teaching Hospital outpatient clinic;

† a patient who presented with two types of suspected adverse drug reactions (dystonia and hypothermia) to more than one medicine;

‡ a patient who presented with two types of suspected adverse drug reactions (electrolyte disturbances and hyperglycemia) to a single suspected medicine;

§ self-medicated medicine (medicine not prescribed by a doctor);

|| medicine prescribed at a primary healthcare center;

¶ medicine prescribed by a general practitioner. URTI = upper respiratory tract infection.

**Table 6.** Clinical details and characteristics of inpatients who experienced adverse drug reactions (ADR) and the suspected medicines

Adverse drug reactions	Admission diagnosis	Medicine(s) administered	Suspected medicine(s)	Age	Sex	Severity	Duration of admission
Anaphylactic shock	Retinoblastoma	Vincristine, etoposide, allopurinol	Etoposide	3.5 years	Male	Severe	8 weeks
Anaphylactic shock	Septicemia	Ceftriaxone, gentamicin, artemether/lumefantrine	Ceftriaxone	8.5 years	Female	Severe	3 weeks
Anemia	Recurrent generalized seizure	Carbamazepine, artemether/lumefantrine	Carbamazepine	4 years	Male	Severe	4 weeks
Angioneurotic edema	Lobar pneumonia	Amodiaquine, cefuroxime, gentamicin	Amodiaquine	10 years	Female	Severe	4 weeks
Tachycardia	Acute severe asthma	Salbutamol, prednisolone, erythromycin	Salbutamol	10.5 years	Male	Moderate	1 week
Constipation	Painful crises in a sickle cell anemic patient	Tramadol, gentamicin, cefuroxime, artemether/lumefantrine	Tramadol	6 years	Female	Moderate	2 weeks
Constipation	Amoebiasis	Metronidazole, chloroquine, amoxicillin	Metronidazole, amoxicillin	8 years	Male	Moderate	2 weeks
Constipation	Osteomyelitis	Pentazocine, ibuprofen, vancomycin, ciprofloxacin	Pentazocine	6 years	Female*	Moderate	6 weeks
Sedation	Osteomyelitis	Pentazocine, ibuprofen, vancomycin, ciprofloxacin	Pentazocine	6 years	Female*	Moderate	6 weeks
Diarrhea	Presumed sepsis	Amoxicillin/clavulanic acid, gentamicin, paracetamol	Amoxicillin/clavulanic acid	3 years	Female	Moderate	2 weeks
Dystonia	Malaria	Chloroquine, promethazine, paracetamol	Promethazine	12 years	Male	Moderate	1 week
Erythema multiforme	Cerebral abscess	Cefotaxime, Ampicillin, phenobarbitone	Ampicillin, phenobarbitone	0.5 years	Male	Severe	6 months
Erythema multiforme	Presumed sepsis	Ciprofloxacin, Cefixime	Cefixime	9 years	Male*	Severe	4 weeks
Seizure	Presumed sepsis	Ciprofloxacin, Cefixime	Cefixime	9 years	Male*	Severe	4 weeks
Electrolyte disturbances	Nephrotic syndrome	Frusemide, potassium supplement, prednisolone, ciprofloxacin	Frusemide, prednisolone	11 years	Male	Moderate	4 weeks
Electrolyte disturbances	Congestive cardiac failure	Frusemide, potassium supplement (under dosage), crystalline penicillin	Frusemide	10 years	Male*	Moderate	2 weeks
Ileus	Congestive cardiac failure	Frusemide, potassium supplement (under dosage), crystalline penicillin	Frusemide	10 years	Male*	Moderate	2 weeks
Hemolysis	Malaria	Co-trimoxazole artemether/lumefantrine, paracetamol	Co-trimoxazole	10 years	Male	Severe	4 weeks
Transient loss of vision	Malaria	Quinine, paracetamol	Quinine	6 years	Male	Moderate	4 weeks
Macular and morbiliform rash	Presumed sepsis	Cefotaxime, gentamicin	Cefotaxime	5 days	Male	Moderate	3 weeks
Pustular rash	Sepsis	Vancomycin, gentamicin	Vancomycin	3 days	Female	Moderate	2 weeks
Pustular rash	Sepsis	Vancomycin, gentamicin	Vancomycin	2 days	Female	Moderate	2 weeks
Pustular rash	Sepsis	Vancomycin, gentamicin	Vancomycin	2 days	Female	Moderate	4 weeks
Pustular rash	Sepsis	Vancomycin, gentamicin	Vancomycin	4 days	Male	Moderate	4 weeks
Red man syndrome	Osteomyelitis	Vancomycin, chloramphenicol	Vancomycin	3 years	Male	Severe	6 weeks
Red man syndrome	Osteomyelitis	Vancomycin, chloramphenicol	Vancomycin	6 years	Female	Severe	6 weeks

* Patients manifesting two types of adverse drug reactions to a single medicine (pentazocine, cefixime or frusemide).

### Causality and avoidability of ADRs

Because many of the patients used multiple numbers of medicines with potentials for ADRs, we did causality assessment for each of the suspected medicines. Nine ADRs were considered definite, 18 probable and 26 possible. Only eight ADRs were judged to be avoidable: electrolyte disturbance and hyperglycemia due to prolonged use of prednisolone; erythema multiforme due to prolonged use of phenobarbitone; electrolyte disturbance and ileus due to prolonged use of frusemide; hemolysis due to the use of co-trimoxazole in a glucose-6-phosphatase dehydrogenase deficient patient; and red man syndrome due to rapid administration of intravenous vancomycin (Tables 5 and 6). Out of the 12 patients admitted due to ADRs (Table 5), three were due to the use of non-prescribed medicines (parental self-medication). Type A accounted for 33 (82.5%) of the ADRs while the remaining seven (dystonia due to amodiaquine, artesunate or cefixime; hypothermia due to amodiaquine, artesunate or cefixime; macular and morbiliform rash due to albendazole; anemia due to carbamazepine; angioneurotic edema due to amodiaquine; seizure due to cefixime; and transient loss of vision due to quinine) were idiosyncratic.

### Case description of ADRs

Fifteen ADRs (37.5%) were severe and 23 (57.5%) were moderate (Tables 5 and 6). ADRs were the cause of death in two fatal cases. Most of the survivors recovered without long-term sequelae. The two deaths resulted from Stevens-Johnson syndrome (SJS). Erythema multiforme was the most frequent form of severe ADR manifested by the patients. The two most frequently experienced moderate ADRs were pustular rashes (4) from the use of vancomycin, and macular and morbiliform rashes (3) from the use of ampicillin, albendazole and cefotaxime.

### Direct medical costs of ADRs to the hospital

The mean costs per moderate and severe ADR to the hospital are reported in Table 2. The mean total direct cost of moderate ADRs was significantly lower than for severe ADRs (₦ 15,317.32 ± 1,882.61; USD 129.81 ± 15.95 versus ₦ 71,893.53 ± 16,520.10; USD 609.27 ± 140.00; P < 0.001).

### Direct medical costs, direct non-medical costs and indirect costs of ADRs to patients

The mean total direct costs to parents for treating moderate and severe ADRs are summarized in Table 7. The total direct cost is the sum of the direct medical costs, direct non-medical costs and indirect costs of ADRs. The cost incurred by the parents for treating severe ADRs was significantly higher than the cost of treating moderate ADRs (₦ 162,713.00 ± ₦ 32,422.00; USD 1,376.92 ± 274.76 versus ₦ 21,246.00 ± ₦ 1,786.00; USD 180.05 ± 15.13; P < 0.001).

**Table 7.** Mean cost to a single patient per adverse drug reaction (ADR) admission in 2007 naira

Direct medical and non-medical costs to patients	Moderate (n = 5)	Severe (n = 7)	P-value
Direct non- medical costs*	₦ 5,400.00 ± ₦ 315.00	₦ 42,308.00 ± ₦ 8,921.00	< 0.001
Private hospital visit cost†	₦ 2,240.00 ± ₦ 140.00	₦ 38,450.00 ± ₦ 7,720.00	< 0.001
Medical costs during admission at LASUTH‡	₦ 3,448.00 ± ₦ 216.00	₦ 46,890.00 ± ₦ 9,120.00	< 0.001
Follow-up visit costs§	₦ 6,315.00 ± ₦ 422.00	₦ 16,225.00 ± ₦ 3,115.00	< 0.001
Total direct costs	₦ 17,403.00 ± ₦ 1,093.00 (USD 147.48 ± 9.26)	₦ 143,873.00 ± ₦ 28,816.00 (USD 1,217.26 ± 244.20)	< 0.001
Indirect medical costs||	₦ 3,843.00 ± ₦ 693.00 (USD 32.57 ± 5 .87)	₦ 18,840,00 ± ₦ 3,606.00 (USD 159.66 ± 30.56)	< 0.001
Total cost of an ADR to parents	₦ 21,246.00 ± ₦ 1,786.00 (USD 180.05 ± 15 .13)	₦ 162,713.00 ± ₦ 32,422.00 (USD 1, 376.92 ± 5274.76)	< 0.001
Estimated annual income	₦ 210,825.00 ± ₦ 15,225.70 (USD 1,786.65 ± 129.03)	₦ 186,608.00 ± ₦ 32,318.44 (USD 1,581.42 ± 273.88)	0.693
% of annual income	10.08%	87.20%	< 0.001

n = number of patients with ADRs. ₦ = 2007 naira (Nigerian currency). USD = United States dollar.

* the sum of all transportation, food and hotel expenses incurred during hospital admission for ADRs;

† expenses incurred on medications, investigations and procedures during initial visit to a private hospital;

‡ expenses incurred on medications, investigations and procedures during admission at LASUTH;

§ expenses incurred on medications, investigations and procedures during follow up visits to LASUTH or a private hospital;

|| lost by each parent, caused by nursing a child with an ADR.

### Total cost of treating ADRs

The mean cost per ADR treatment is presented in Table 8. The mean treatment cost per severe ADR was significantly higher than the cost per moderate ADR (₦ 234,606.00 ± ₦ 48,942.10; USD 1,988.19 ± 414.76 versus ₦ 36,563.32 ± ₦ 3,668.61; USD 309.86 ± 31.09; P < 0.001).

**Table 8.** Mean cost per adverse drug reaction (ADR) treatment in 2007 naira

Parameters	Moderate (n = 5)	Severe (n = 7)	P-value
Hospital expenditure (Government perspective)	₦ 15,317.32 ± ₦ 1,882.61	₦ 71,893.53 ± ₦ 16,520.10	< 0.001
Parents’ expenditure (Patients’ perspective)	₦ 21,246.00 ± ₦ 1,786.00	₦ 162,713.00 ± ₦ 32,422.00	< 0.001
Total cost of treatment (Society's perspective)	₦ 36,563.32 ± ₦ 3,668.61 (USD 309.86 ± 31.09)	₦ 234,606.00 ± ₦ 48,942.10 (USD 1,988.19 ± 414.76)	< 0.001

n = number of patients with ADRs; ₦ = in 2007 naira (Nigerian currency); USD = United States dollar.

## DISCUSSION

It is evident from this study that ADRs in children pose a financial burden both to parents and to hospitals in Nigeria. The overall incidence (1.8%) of ADRs in this study is comparable with the 1.1% previously reported in Nigeria.^3^ However, when this rate is compared with results (2.1% to 9.5%) from developed countries,^6,7^ ADRs are still grossly underreported in Nigeria.

More males than females were affected with ADRs, probably because more males than females were admitted over the study period. Moreover, parents tend to seek healthcare earlier for male than for female children,^31^ probably due to male child preference.^32^ The proportion of children admitted due to suspected ADRs (0.6%) was half the proportion who developed ADRs while on admission (1.2%). This disparity may be as a result of the exclusion of mild ADRs from the admissions. Parents in Nigeria traditionally consider skin manifestation of ADRs as measles rash. Measles is usually perceived as a punishment for breaking family taboos or as an evil deed from witches or enemies.^33^ Parents may therefore not seek modern medical care for children with measles or skin manifestation of ADRs, which might have invariably contributed towards the low proportion of children admitted due to suspected moderate and severe ADRs.

One study has reported poor perceptions of ADR reporting among doctors working in a teaching hospital in Nigeria.^34^ At LASUTH, only doctors and nurses usually participate in ward rounds and they are likely to underreport ADRs. Previous studies showed that many doctors do not record all the symptoms of ADRs reported to them by adult patients.^35^ Therefore, a multidisciplinary approach towards ADR surveillance, as practiced in this study, may be necessary for effective pediatric pharmacovigilance in Nigeria.

We have previously reported that antibiotics and antimalarials are associated with ADRs in children.^2^ This trend has remained unchanged in the present study, since antibiotics and antimalarials remained the most frequently incriminated medicines in ADRs. Thus, it appears that infectious diseases are still a menace to children in Lagos, Nigeria.

The most common organs and systems associated with ADRs in this study were the skin and the circulatory system (Table 4). Previous studies equally identified dermatological manifestations as the most common presentation of ADRs in children^29^ and adults.^36^ Erythema multiforme was the most frequent skin manifestation of ADR in this study, and was associated with antibiotics (ampicillin and co-trimoxazole), antimalarials (sulfadoxine/pyrimethamine) and anticonvulsants (phenobarbitone). The association between these medicines and erythema multiforme had been reported earlier.^2^

Polypharmacy was practiced by many of our patients, and more than one medicine was often suspected to be the cause of the ADRs. We therefore undertook causality assessment for the individual medicine using the Jones method.^28^ In order to improve the accuracy of our assessments, two expert members of the research team undertook the causality assessment, which yielded 70% agreement. Any disagreements were resolved by consensus discussions with other members of the team. Causality assessments are difficult, and inter-rater agreements may vary.^37^ It was impossible for us to ascertain definite causality between the ADRs and many of the suspected medicines because this requires re-challenge: a procedure that the parents would not consent to. However, the majority of the ADRs were suspected to be probably based on de-challenge. This limitation therefore highlights the special challenge that faces ADR assessment in children.

Unlike the previous studies in adult populations, in which most of the ADRs were avoidable,^38^ only 20% of the ADRs observed in this study were avoidable, which is consistent with the proportion reported in another pediatric population by Weiss et al.^6^ The government may therefore need to review the policy on sales and use of pediatric medicines in Nigeria, which should be complemented with development and implementation of preventive strategies against ADRs. Given the wide variety of medicines implicated and the array of ADRs identified in this study, affecting many organ systems in the body, prevention is likely to require complex multifaceted intervention strategies. Computerized prescribing and monitoring systems^39^ have been adopted in developed countries to prevent ADRs, but may be too expensive to implement in Nigeria. However, less expensive methods like participation of pharmacists in ward rounds,^40^ drug treatment monitoring,^41^ and enhanced education and training in prescribing^42^ can be implemented in Nigeria to reduce the burden of ADRs.

Previous studies that looked into the estimated cost of ADRs in developed countries focused only on adult populations, and estimates were mainly based on the cost of hospitalizing the patients.^10,11^ Contrarily, in the developing countries where attempts had been made to estimate the cost of ADRs, pediatric children were studied along with adults. The direct medical and direct non-medical costs to the patients were not considered in previous studies,^43,44^ thus underestimating the financial burden of ADRs. However, the present study focused on a pediatric population and estimated the direct and indirect medical costs as well as the non-medical costs to both the hospital and the parents.

Among the estimated medical expenditures, the mean costs of hospitalization for moderate ADRs (₦ 2,080.00 ± ₦ 420.00; USD 17.63 ± 3.56) and severe (₦ 3,140.00 ± ₦ 680.00; USD 26.61 ± 5.76) were relatively cheaper than the costs reported in previous studies from developed countries^10,11^ and other developing countries.^12^ The differences in the cost may be attributed to the high level of poverty in Nigeria and lack of sophisticated and advanced technology to investigate and monitor patients with ADRs. Similarly, a high cost of hospitalization, similar to the costs reported in previous studies,^10-12^ may discourage patients with ADRs from seeking hospital care.

The total estimated cost per moderate and per severe ADR were ₦ 36,563.32 ± ₦ 3,668.61 (USD 309.86 ± 31.09) and ₦ 234,606.00 ± ₦ 48,942.10 (USD 1,988.19 ± 414.76) respectively, which are five to thirty times the minimum monthly wage (₦ 7,500 = USD 63.56) of an average worker in Lagos. The mean annual income of the parents ranged from ₦ 186,608.00 ± ₦ 32,318.44 (USD 1581.42 ± 273.89) to ₦ 210,825.00 ± ₦ 15,225.70 (USD 1786.65 ± 129.03), thus indicating that the parents were from low socioeconomic classes.^45^ Expending 10.8% to 87.20% of their annual income to treat just one hospitalized case of moderate to severe ADRs is worrisome. Thus, these data indicate that the treatment of ADRs may place a large financial burden on Nigerian households. If pediatric health care were completely free, the hospital would have expended approximately 1.83 million naira (USD 15,466.60) to treat the 12 patients admitted due to ADRs over an 18-month period. This sum of money is staggering and represents a significant financial burden on the government, especially in a resource-poor country.

The fourth Millennium Development Goal (MDG4) is to reduce child mortality by two-thirds between 1990 and 2015 through prevention and adequate treatment of common killer childhood diseases.^46^ These diseases include pneumonia, diarrhea, malaria, measles and meningitis. In spite of the efforts of the Nigerian government to achieve this goal, children are still dying of these killer diseases in high numbers.^47^ The mean cost estimates for malaria and meningitis during hospital admission, over the 18-month period, were not determined. However, a mean cost per admission in the range of USD 47 to USD 96 and USD 54 to USD 285 has been reported in Kenyan public regional and national hospitals for treating malaria and meningitis, respectively.^48^ These cost estimates included the cost of hospitalization and may be extrapolated to the treatment cost for malaria and meningitis in Nigeria because of the similarities in the gross domestic product (GDP) of the two countries. Considering the high proportion of children who were afflicted with malaria and meningitis in this study, and the potential risks of morbidity and mortality that may complicate these diseases, it will be more reasonable for the government to direct the large sum of money expended on the few cases of ADRs to fight the killer childhood diseases and implement preventive measures against ADRs.

In this study, we were unable to statistically determine the risk factors associated with ADRs due to the small number of cases with suspected ADRs. It would have been more appropriate to compare the mean estimated cost of suspected ADRs with the mean cost of treating non-ADR patients while on admission, or to compare the estimated cost of ADRs with the proportion of total annual hospitalization costs for all the children. However, lack of access to detailed information on all the non-ADR patients made this impossible. We compared the cost estimates of ADRs in children with those of adults because of a lack of data on children. This therefore calls for caution in interpreting our results. Another limitation of this study is that the indirect costs involving the lost work days and productivity cost to the parents due to morbidity and mortality of the patients were not included in the cost estimates. Lack of records of the yearly population of pediatric patients who developed ADRs in the hospital has also made it impossible for us to calculate the annual cost of treating ADRs.

It is very likely that we have underestimated the financial burden of ADRs because all the patients were managed in the open wards, instead of managing the severe and fatal cases in a pediatric intensive care unit. Some very important investigations such as arterial blood gas levels were not done due to lack of facilities. The costs of these facilities and investigations, if available, may be very high. The exclusion of mild cases of suspected ADRs and the inpatients who developed ADRs might also have contributed towards the underestimation of the financial burden of ADRs in this study.

ADRs are a significant public health problem that is associated with prolonged hospitalization, morbidity and mortality. In spite of a number of methodological limitations, this study has provided data for future reference to the estimated cost of ADRs in children. The cost of treating ADRs in children was substantial and imparted a significant financial burden on both the parents and the hospital. Public enlightenment campaigns on rational use of medicines, strengthening of policies on the sale and use of prescribed medicines, promotion of pharmacoeconomic studies and cooperation between clinicians, clinical pharmacologists and pharmacists in pharmacovigilance remain the cornerstone for preventing ADRs and decreasing their costs. More efforts should be devoted to achieving these interventions.

## CONCLUSION

The overall incidence of ADRs as a cause of pediatric hospital admissions was 1.8%, and the age of these patients was not different from the non-ADR inpatients. The incidence after admission was 1.2%. The mean cost of treating a single case of severe ADR (₦ 234,606.00 ± ₦ 48,942.10; USD 1,988.19 ± 414.76) was significantly higher than the cost for a moderate (₦ 36,563.32 ± ₦ 3,668.61; USD 309.86 ± 31.09) ADR (P < 0.001).
